# Data of microwave assisted extraction and conventional hot water extraction of *Dendrobium* Sonia ‘Earsakul’ orchid flower

**DOI:** 10.1016/j.dib.2020.105906

**Published:** 2020-06-21

**Authors:** Siriyupa Netramai, Thitisilp Kijchavengkul, Hayati Samsudin, Sittiwat Lertsiri

**Affiliations:** a School of Bioinnovation and Bio-based Product Intelligence, Faculty of Science, Mahidol University, Nakhon Pathom 73170, Thailand; b School of Industrial Technology, Universiti Sains Malaysia, Penang 11800, Malaysia; c Department of Biotechnology, Faculty of Science, Mahidol University, Bangkok 10400, Thailand

**Keywords:** Microwave assisted extraction, Hot water extraction, Dendrobium Sonia ‘Earsakul’, Dendrobium hybrid, UV spectrophotometry, Anthocyanin, RSM

## Abstract

Crude extracts of fresh *Dendrobium* Sonia ‘Earsakul’ orchid flowers (DSE) were prepared using microwave assisted extraction (MAE; using household microwave oven) and hot water extraction (HWE; at constant 80 °C). The obtained DSEs were measured their absorbance at *λ_max_* of 543 and 583 nm and determined their total monomeric anthocyanin contents (TAC). Mathematical models of MAE of *Dendrobium* Sonia ‘Earsakul’ orchid flower were constructed using response surface methodology - Box-Behnken design. Studied parameters included flower to water ratio, microwave power, and extraction time, with absorbance at *λ_max_* as response. The data generated were 1) visible spectrum (400–700 nm) of DSE; 2) absorbance values at *λ_max_* and 3) TAC of DSEs obtained from various extraction conditions of MAE and HWE; 4) linear equations describing correlations between TAC and absorbance at *λ_max_* of DSEs; and 5) mathematical models of MAE of *Dendrobium* Sonia ‘Earsakul’ orchid.

Specifications Table

**Table:** 

**Subject**	Agricultural and Biological Sciences (General)
**Specific subject area**	Extraction of crude pigments from flower
**Type of data**	Table Figure
**How data were acquired**	Microwave assisted extraction (LG MG-3937C Microwave Oven, LG Electronics, Bangkok, Thailand) Hot water extraction (Memmert Waterbath WNE 22, Schwabach, Germany, set at 80 °C) Spectrophotometry (Shimadzu UV-1280 UV/Vis Spectrophotometer, Bara Scientific Co., Ltd., Thailand) Total monomeric anthocyanin pigment content (pH differential method) Statistical analysis (JMP 8.0 program, SAS Institute Inc., NC, USA)
**Data format**	Raw Analyzed
**Parameters for data collection**	Parameters for response surface methodology of microwave assisted extraction were flower to water ratio (1:5 to 1:3 g/ml), microwave power (480 to 800 W), and extraction time (2 to 8 min); and response was absorbance at $\lambda_{max}$. Parameter for hot water extraction (at constant 80 °C and flower to water ratio of 1:3 g/ml) was extraction time (10 to 180 min, with increment of 10 min). Efficacies of microwave assisted extraction (MAE) and hot water extraction were assessed through absorbance at $\lambda_{max}$ (543 and 583 nm)
**Description of data collection**	Microwave assisted extraction (MAE) and hot water extraction were used to prepare crude extracts from fresh *Dendrobium* Sonia ‘Earsakul’ orchid flowers. Response surface methodology (Box-Behnken design) was used to generate extraction conditions for MAE. The extracts were measured their absorbance of visible light (400–700 nm) and total monomeric anthocyanin pigment content, using pH differential method. The experiment was conducted in triplicate.
**Data source location**	Purchase of fresh *Dendrobium* Sonia ‘Earsakul’ orchid flowers: Taling Chan, Bangkok, Thailand 13°45′14.7″N 100°26′38.7″E 13.754088, 100.444088 Research experiment and data analysis: Salaya, Nakhon Pathom, Thailand 13°47′33.5″N 100°19′21.2″E 13.792907, 100.322589
**Data accessibility**	With the article

## Value of the Data

This data set of spectrophotometric properties of crude aqueous extract of *Dendrobium* Sonia ‘Earsakul’ orchid flower can be used as reference for optical properties of potential alternative natural dye for both food and non-food applications.
The data on mathematical modeling of microwave-assisted extraction of *Dendrobium* Sonia ‘Earsakul’ can serve as preliminary data for aqueous extraction of purple Dendrobium orchids prepared for developments of textiles, foods and beverages, or household chemicals.
The data of total monomeric anthocyanin content of crude aqueous extract of *Dendrobium* Sonia ‘Earsakul’ contributes in building database of anthocyanin-rich plants.

## 1 Data description

**Fig. 1:**
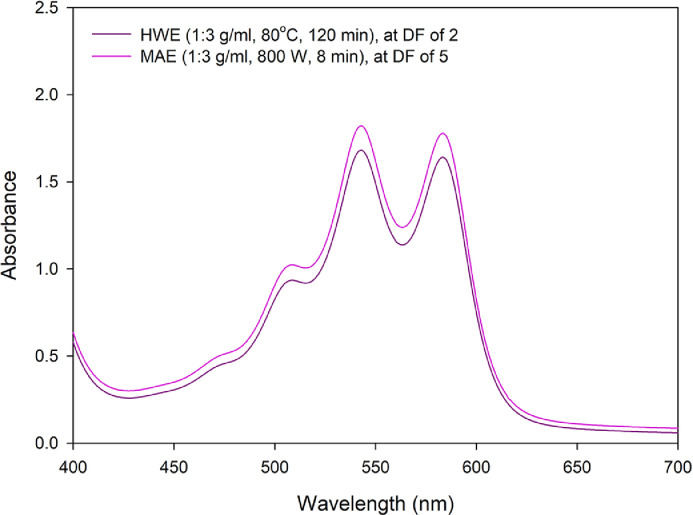
Visible spectra of crude color extract of fresh *Dendrobium* Sonia ‘Earsakul’ orchid flowers obtained through MAE and HWE; DF = 5 for extract obtained through MAE and 2 for extract obtained through HWE.

Fig. 1 and Supplemental data 1 report visible spectra (400–700 nm) of crude color extract from fresh *Dendrobium* Sonia ‘Earsakul’ orchid flowers (DSE) obtained through microwave assisted extraction (MAE; flower to water ratio of 1:3 g/ml, microwave power of 800 W, extraction of 8 min) and hot water extraction (HWE; flower to water ratio of 1:3 g/ml, constant 80 °C, extraction time of 120 min). Solvent used was distilled water. Note the difference in dilution factor (DF) of both crude extracts, *i.e.* 5 and 2 for MAE and HWE, respectively.

Table 1 shows linear equations outlining correlations between absorbance at *λ_max_* of 543 and 583 nm and the corresponding total monomeric anthocyanin contents (TAC) of DSE. The data included in the analysis belongs to those obtained through MAE method (Supplemental data 2). The predicted total monomeric anthocyanin content of crude extract (cyd-3-glu equivalents, mg/L) can be converted into predicted TAC per weight (cyd-3-glu equivalents, mg/g or mg/kg) of dried orchid flower using data of moisture content wet basis (%) of fresh *Dendrobium* Sonia ‘Earsakul’ orchid flower supplied in Supplemental data 3.

**Table 1:** Linear equations of correlations between total monomeric anthocyanin content and absorbance at *λ_max_* of *Dendrobium* Sonia ‘Earsakul’ orchid crude extracts obtained through MAE.

*λ_max_* (nm)	Predictive model*	R^2^
543	y=4.9952x+3.5347	0.8612
583	y=5.1910x+3.4738	0.8626

⁎ y represents total monomeric anthocyanin content (cyd-3-glu equivalents, mg/L) of crude extract and *x* is absorbance at *λ_max_* of the extracts.

**Fig. 2:**
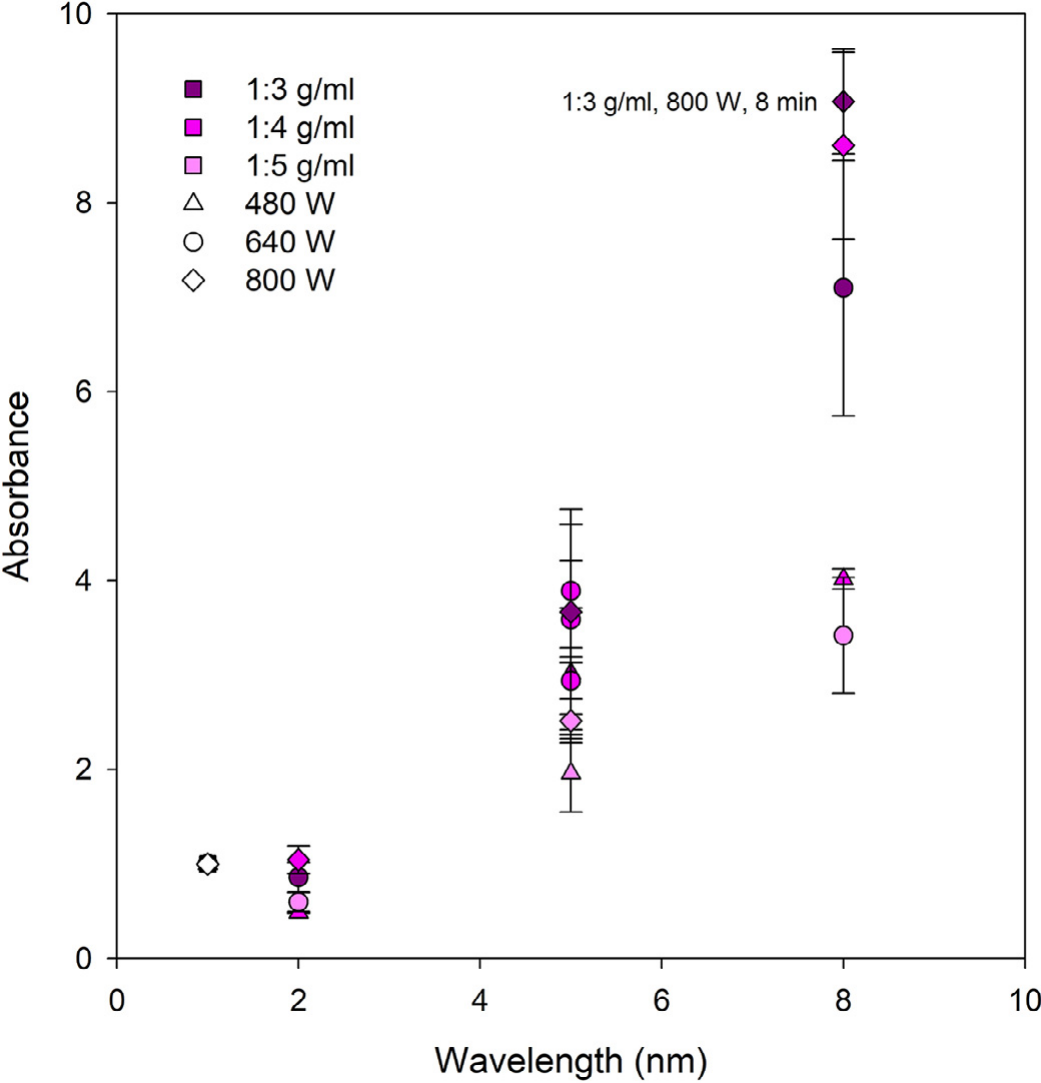
Absorbance at *λ_max_* of 543 nm of *Dendrobium* Sonia ‘Earsakul’ orchid crude extracts obtained through MAE.

**Fig. 3:**
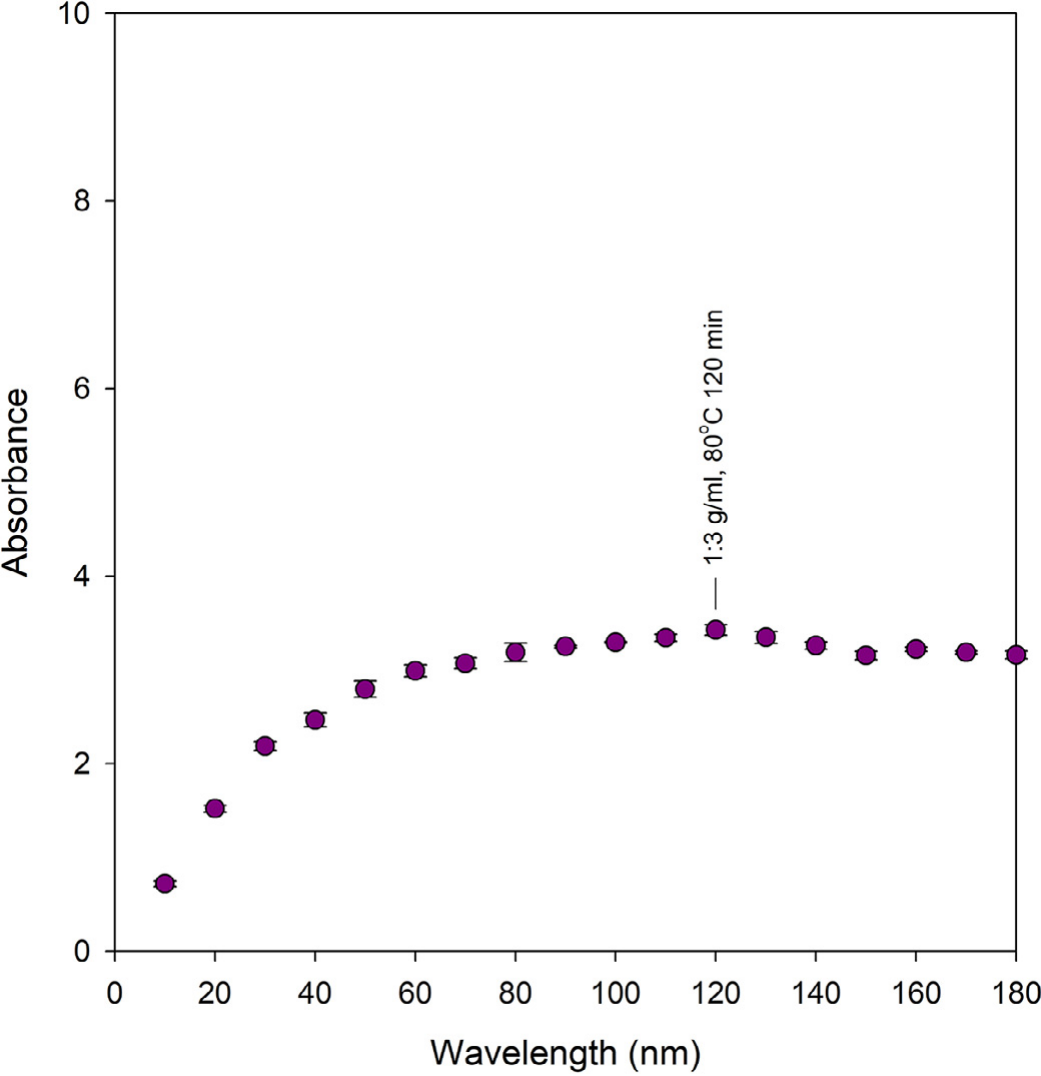
Absorbance at *λ_max_* of 543 nm of *Dendrobium* Sonia ‘Earsakul’ orchid crude extracts obtained through HWE.

Fig. 2, Fig. 3 show absorbance at *λ_max_* of 543 nm of DSEs obtained using MAE (flower to water ratio of 1:5 to 1:3 g/ml, microwave power of 480–800 W, extraction time of 2–8 min) and HWE (flower to water ratio of 1:3 g/ml, constant 80 °C, 10–180 min), respectively (Supplemental data 2). Solvent used was distilled water.

Table 2 shows parameter estimates of mathematical models for MAE of *Dendrobium* Sonia ‘Earsakul’ orchid. The predictive models had R^2^ of 0.9432 and 0.9407 for data collected at *λ_max_* of 543 and 583 nm, respectively (Supplemental data 4).

**Table 2:** Parameter estimates of RSM equations for MAE of *Dendrobium* Sonia ‘Earsakul’ orchid.

Parameter	*λ_max_* = 543 nm			*λ_max_* = 583 nm		
	Estimate	T Ratio	Prob > | t |	Estimate	T Ratio	Prob > | t |
Intercept	−3.902207	−5.17	<0.0001*	−3.766574	−5.13	<0.0001*
flower to water ratio	0.7689375	4.71	<0.0001*	0.7331167	4.62	<0.0001*
Microwave power	0.0049633	4.87	<0.0001*	0.0048103	4.85	<0.0001*
Extraction time	0.8395569	15.44	<0.0001*	0.8079181	15.28	<0.0001*
Ratio*(Power-640)	0.0001494	0.10	0.9181	0.0001043	0.07	0.9411
Ratio*(Time-5)	0.2846306	3.70	0.0007*	0.2705056	3.62	0.0009*
(Power-640)*(Time-5)	0.0020987	4.37	0.0001*	0.0020262	4.34	0.0001*
Ratio*Ratio	−0.613793	−2.56	0.0151*	−0.599779	−2.57	0.0146*
(Power-640)*(Power-640)	−2.656e-6	−0.28	0.7788	−1.928e-6	−0.21	0.8338
(Time-5)*(Time-5)	0.0149045	0.56	0.5800	0.013844	0.53	0.5969

⁎ indicates significance of the effects at type I error (*α*) of 0.05.

## 2 Experimental design, materials, and methods

### 2.1 Materials and reagents

**Fig. 4:**
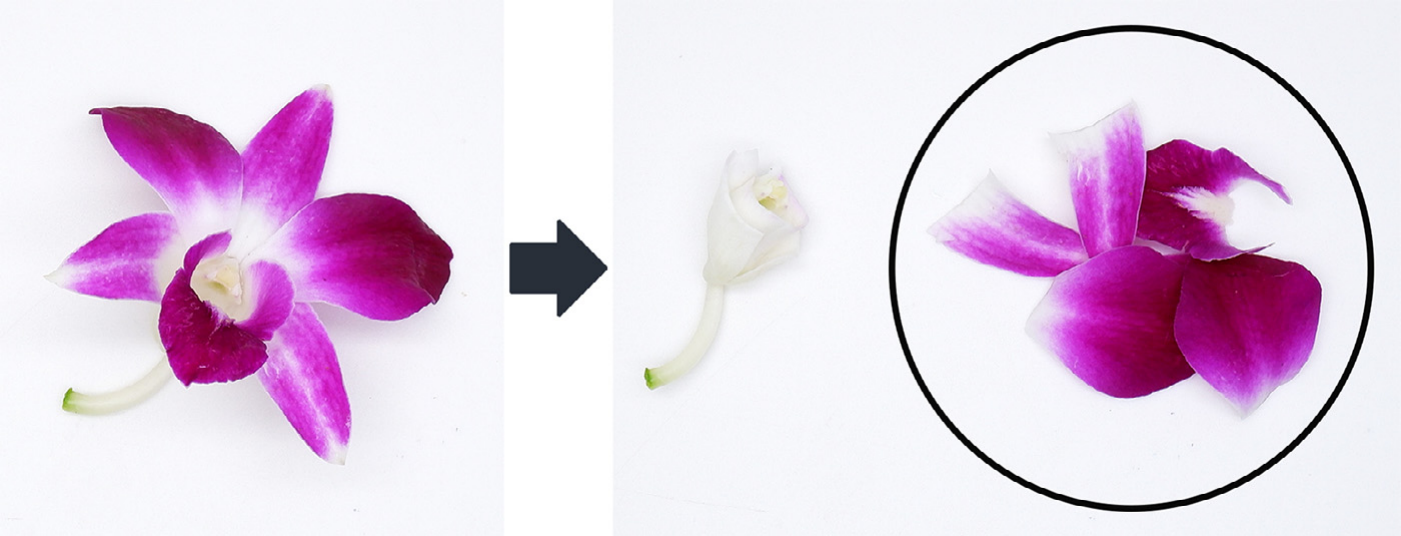
Parts of fresh *Dendrobium* Sonia ‘Earsakul’ orchid flowers utilized for extraction (in circle).

Fresh *Dendrobium* Sonia ‘Earsakul’ orchid flowers (growth stage 5) [1] were purchased from local supermarkets in Bangkok, Thailand. Fresh orchids were stored at 4 ± 1 °C until used and used within 3 days of purchase. Flowers were cut into small pieces and only the parts with purple color, *i.e.* majority of petal part, were used (Fig. 4). The cut petals were subjected to extraction process within 1 h of size reduction.

Whatman^Ⓡ^ filter paper No. 1 (Sigma-Aldrich, Inc., St. Louis, MO, USA) was purchased from Business organization of the office of the welfare promotion commission for teachers and educational personnel (BOWT), Bangkok, Thailand. Disposable plastic cuvette (Bibby Scientific Ltd., Staffordshire, UK) was used in UV–Vis spectrophotometry.

For determination of total monomeric anthocyanin pigment content, colorless buffer solutions of pH 1.0 and 4.5 used were prepared from potassium chloride (0.025 M) (KCl, Ajax Finechem, New South Wales, Australia) and sodium acetate (0.4 M) (CH3CO2Na·3H2O, Ajax Finechem), respectively. The buffer solutions were adjusted their final pH with hydrochloric acid (HCl, Fisher Scientific, MA, USA) [2].

### 2.2 Microwave assisted extraction

Prior to extraction, known amount of cut flower pieces were immersed in distilled water at room temperature, for 60 s to ensure thorough submersion. Table 3 shows microwave assisted extraction (MAE) conditions for cut orchid flowers. The extractions were performed in random order according to Box-Behnken design with response surface methodology. All extractions were conducted in triplicate. Household microwave oven (LG MG-3937C Microwave Oven, LG Electronics, Bangkok, Thailand) was used for microwave heating process. After the extractions, the heated mixtures were filtered, and DSEs were collected and left to cool to room temperature [3].

**Table 3:** Conditions for MAE of Fresh *Dendrobium* Sonia ‘Earsakul’ orchid flower*.

Treatment	Code	Flower to water (g/ml)	Microwave power (W)	Extraction time (min)
1	0 0 0	1:4	640	5
2	0 – –	1:4	480	2
3	0 – +	1:4	480	8
4	+ – 0	1:3	480	5
5	0 0 0	1:4	640	5
6	+ 0 –	1:3	640	2
7	+ 0 +	1:3	640	8
8	0 0 0	1:4	640	5
9	– – 0	1:5	480	5
10	– + 0	1:5	800	5
11	– 0 –	1:5	640	2
12	– 0 +	1:5	640	8
13	0 + –	1:4	800	2
14	0 + +	1:4	800	8
15	+ + 0	1:3	800	5

⁎ Extraction condition of flower to water ratio of 1:3 g/ml, microwave power of 800 W, and extraction time of 8 min (+ + +) was not included as treatment according to RSM-Box-Behnken design; the extraction was done additionally and the data was list in Fig. 2 and Supplemental data 2 along with those obtained using extraction conditions list in the table.

### 2.3 Hot water extraction

Freshly cut orchid flowers were submerged in 80 °C distilled water (flower to water ratio of 1:3 g/ml) placed in temperature-controlled water bath (Memmert Waterbath WNE 22, Schwabach, Germany), for 10 to 180 min (in increment of 10 min). The heated mixtures were filtered through Whatman filter paper No. 1. The filtrates were collected and used as DSEs, and the plant residues were discarded. DSEs were left to cool to room temperature before further testing. All experiments were performed in triplicate.

### 2.4 UV–Vis spectroscopy

To obtain DSEs’ visible spectra (400–700 nm), *λ_max_*, and absorbance at *λ_max_*, UV–Vis spectrophotometer (Shimadzu UV-1280 UV/Vis Spectrophotometer, Bara Scientific Co., Ltd., Thailand) was used. The measurements were conducted within 1 h of extractions.

### 2.5 Determination of total monomeric anthocyanin pigment content

Total monomeric anthocyanin pigment content in DSEs was determined according to pH differential method [2]. The extracts were mixed with pH 1.0 or pH 4.5 buffer solutions for final concentration of 10% vol/vol, and then the mixtures were measured their absorbance at 520 and 700 nm, using UV–Vis spectrophotometer. The anthocyanin pigment content, as cyanidin-3-glucoside, was calculated as described in Eq. (1) and 2.

(1) $$A={\left(A_{520}-A_{700}\right)}_{\text{pH}1.0}-{\left(A_{520}-A_{700}\right)}_{\text{pH}4.5}$$

Total monomeric anthocyanin (cyanidin-3-glucoside equivalents, mg/L)

(2) $$=\left(A\times M_{w}\times \text{DF}\times 10^{3}\right)/\left(\varepsilon \times l\right)$$

where *M_w_* is molecular weight of cyanidin-3-glucoside (cyd-3-glu) (449.2 g/mol); DF is dilution factor; *ι* is pathlength in cm (1 cm); ɛ is molar extinction coefficient for cyd-3-glu (26,900 L·mol^−1^·cm^−1^).

### 2.6 Determination of moisture content

To determine moisture content wet basis (%) of fresh *Dendrobium* Sonia ‘Earsakul’ orchid flower, oven-drying method outlined by AOAC (Official Method 935.29) was used [4]. Drying temperature applied was 103 °C (France Etuves XU112, Merit Tech Co., Ltd., Thailand).

### 2.7 Statistical analysis

All data obtained were statistically analysed using JMP 8.0 program (SAS Institute Inc., NC, USA) at the confidence level of 95% (*α* = 0.05) with Tukey's adjustment for comparison of the means.

Absorbance at *λ_max_* of crude extracts were used to construct predictive models (Eq. (3)) for MAE of *Dendrobium* Sonia ‘Earsakul’ orchid, using JMP 8.0 program.

(3) $$y=\beta_{0}+\beta_{1}x_{1}+\beta_{2}x_{2}+\beta_{3}x_{3}+\beta_{12}x_{1}x_{2}+\beta_{13}x_{1}x_{3}+\beta_{23}x_{2}x_{3}+\beta_{11}x_{1}^{2}+\beta_{22}x_{2}^{2}+\beta_{33}x_{3}^{2}+\varepsilon$$

where *y* is absorbance at *λ_max_; x*_1_ is coded values of flower to water ratio (Table 3); *x*_2_ is microwave power (W); *x*_2*c*_ is microwave power – 640 (W); *x*_3_ is extraction time (min); *x*_3*c*_ is extraction time – 5 (min); *β*_0_ is intercept; *β*_1_, *β*_2_, and *β*_3_ are linear effects of flower to water ratio, microwave power, and extraction time, respectively; *β*_11_, *β*_22_, and *β*_33_ are quadratic effects of flower to water ratio, microwave power, and extraction time, respectively; *β*_12_, *β*_13_, and *β*_23_ are interaction effects of flower to water ratio and microwave power, flower to water ratio and extraction time, and microwave power and extraction time, respectively; and ɛ is residual error.

## Declaration of Competing Interest

The authors declare that they have no known competing financial interests or personal relationships which have, or could be perceived to have, influenced the work reported in this article.

## Appendix Supplementary materials

Image, application 1

Image, application 2

Image, application 3

Image, application 4
