# Supplementary material for: Data of microwave assisted extraction and conventional hot water extraction of Dendrobium Sonia ‘Earsakul’ orchid flower
Source: Data Brief. 2020 Jun 21;31:105906. doi: 10.1016/j.dib.2020.105906 (PMC7329924; doi:10.1016/j.dib.2020.105906)
Supplement: Supplementary file 4 [file mmc4.doc]

**Least Squares Fit**

**Response V 543**

**Whole Model**

**Actual by Predicted Plot**

Summary of Fit

|  |  |
| --- | --- |
| RSquare | 0.902554 |
| RSquare Adj | 0.877496 |
| Root Mean Square Error | 0.799211 |
| Mean of Response | 3.180038 |
| Observations (or Sum Wgts) | 45 |

Analysis of Variance

| **Source** | **DF** | **Sum of Squares** | **Mean Square** | **F Ratio** |
| --- | --- | --- | --- | --- |
| Model | 9 | 207.06101 | 23.0068 | 36.0191 |
| Error | 35 | 22.35584 | 0.6387 | **Prob > F** |
| C. Total | 44 | 229.41686 |  | <.0001* |

**Lack o**f Fit

| **Source** | **DF** | **Sum of Squares** | **Mean Square** | **F Ratio** |
| --- | --- | --- | --- | --- |
| Lack of Fit | 3 | 8.487633 | 2.82921 | 6.5282 |
| Pure Error | 32 | 13.868211 | 0.43338 | **Prob > F** |
| Total Error | 35 | 22.355844 |  | 0.0014* |
|  |  |  |  | **Max RSq** |
|  |  |  |  | 0.9396 |

Parameter Estimates

| **Term** |  | **Estimate** | **Std Error** | **t Ratio** | **Prob>|t|** |
| --- | --- | --- | --- | --- | --- |
| Intercept |  | -3.902207 | 0.755463 | -5.17 | <.0001* |
| Ratio |  | 0.7689375 | 0.163138 | 4.71 | <.0001* |
| Power |  | 0.0049633 | 0.00102 | 4.87 | <.0001* |
| Time |  | 0.8395569 | 0.054379 | 15.44 | <.0001* |
| Ratio*Ratio |  | -0.613793 | 0.240133 | -2.56 | 0.0151* |
| Ratio*(Power-640) |  | 0.0001494 | 0.001442 | 0.10 | 0.9181 |
| (Power-640)*(Power-640) |  | -2.656e-6 | 9.38e-6 | -0.28 | 0.7788 |
| Ratio*(Time-5) |  | 0.2846306 | 0.076904 | 3.70 | 0.0007* |
| (Power-640)*(Time-5) |  | 0.0020987 | 0.000481 | 4.37 | 0.0001* |
| (Time-5)*(Time-5) |  | 0.0149045 | 0.026681 | 0.56 | 0.5800 |

Effect Tests

| **Source** | **Nparm** | **DF** | **Sum of Squares** | **F Ratio** | **Prob > F** |  |
| --- | --- | --- | --- | --- | --- | --- |
| Ratio | 1 | 1 | 14.19036 | 22.2162 | <.0001* |  |
| Power | 1 | 1 | 15.13555 | 23.6960 | <.0001* |  |
| Time | 1 | 1 | 152.24887 | 238.3587 | <.0001* |  |
| Ratio*Ratio | 1 | 1 | 4.17314 | 6.5334 | 0.0151* |  |
| Ratio*Power | 1 | 1 | 0.00685 | 0.0107 | 0.9181 |  |
| Power*Power | 1 | 1 | 0.05120 | 0.0802 | 0.7788 |  |
| Ratio*Time | 1 | 1 | 8.74957 | 13.6982 | 0.0007* |  |
| Power*Time | 1 | 1 | 12.17745 | 19.0648 | 0.0001* |  |
| Time*Time | 1 | 1 | 0.19931 | 0.3120 | 0.5800 |  |

**Residual by Predicted Plot**

**Ratio**

**Leverage Plot**

**Power**

**Leverage Plot**

**Time**

**Leverage Plot**

**Ratio*Ratio**

**Leverage Plot**

**Ratio*Power**

**Leverage Plot**

**Power*Power**

**Leverage Plot**

**Ratio*Time**

**Leverage Plot**

**Power*Time**

**Leverage Plot**

**Time*Time**

**Leverage Plot**

Response Surface

| Coef | **Ratio** | **Power** | **Time** | **V 543** |
| --- | --- | --- | --- | --- |
| Ratio | -0.613793 | 0.0001494 | 0.2846306 | 0.7689375 |
| Power | . | -2.656e-6 | 0.0020987 | 0.0049633 |
| Time | . | . | 0.0149045 | 0.8395569 |

Solution

| **Variable** | **Critical Value** |
| --- | --- |
| Ratio | -0.155677 |
| Power | 306.49538 |
| Time | 1.8020744 |

Solution is a

SaddlePoint

Critical values outside data range

Predicted Value at Solution

1.2421905

**Canonical Curvature**

Eigenvalues and Eigenvectors

|  |  |  |  |
| --- | --- | --- | --- |
| Eigenvalue | 0.0456 | -0.0000 | -0.6445 |
| Ratio | 0.21090 | -0.00504 | 0.97749 |
| Power | 0.02281 | 0.99974 | 0.00023 |
| Time | 0.97724 | -0.02225 | -0.21096 |
|  |  |  |  |

**Response V 583**

**Whole Model**

**Actual by Predicted Plot**

Summary of Fit

|  |  |
| --- | --- |
| RSquare | 0.900717 |
| RSquare Adj | 0.875187 |
| Root Mean Square Error | 0.776946 |
| Mean of Response | 3.07188 |
| Observations (or Sum Wgts) | 45 |

Analysis of Variance

| **Source** | **DF** | **Sum of Squares** | **Mean Square** | **F Ratio** |
| --- | --- | --- | --- | --- |
| Model | 9 | 191.67460 | 21.2972 | 35.2809 |
| Error | 35 | 21.12759 | 0.6036 | **Prob > F** |
| C. Total | 44 | 212.80219 |  | <.0001* |

**Lack o**f Fit

| **Source** | **DF** | **Sum of Squares** | **Mean Square** | **F Ratio** |
| --- | --- | --- | --- | --- |
| Lack of Fit | 3 | 8.198901 | 2.73297 | 6.7644 |
| Pure Error | 32 | 12.928689 | 0.40402 | **Prob > F** |
| Total Error | 35 | 21.127591 |  | 0.0012* |
|  |  |  |  | **Max RSq** |
|  |  |  |  | 0.9392 |

Parameter Estimates

| **Term** |  | **Estimate** | **Std Error** | **t Ratio** | **Prob>|t|** |
| --- | --- | --- | --- | --- | --- |
| Intercept |  | -3.766574 | 0.734417 | -5.13 | <.0001* |
| Ratio |  | 0.7331167 | 0.158593 | 4.62 | <.0001* |
| Power |  | 0.0048103 | 0.000991 | 4.85 | <.0001* |
| Time |  | 0.8079181 | 0.052864 | 15.28 | <.0001* |
| Ratio*Ratio |  | -0.599779 | 0.233443 | -2.57 | 0.0146* |
| Ratio*(Power-640) |  | 0.0001043 | 0.001402 | 0.07 | 0.9411 |
| (Power-640)*(Power-640) |  | -1.928e-6 | 9.119e-6 | -0.21 | 0.8338 |
| Ratio*(Time-5) |  | 0.2705056 | 0.074762 | 3.62 | 0.0009* |
| (Power-640)*(Time-5) |  | 0.0020262 | 0.000467 | 4.34 | 0.0001* |
| (Time-5)*(Time-5) |  | 0.013844 | 0.025938 | 0.53 | 0.5969 |

Effect Tests

| **Source** | **Nparm** | **DF** | **Sum of Squares** | **F Ratio** | **Prob > F** |  |
| --- | --- | --- | --- | --- | --- | --- |
| Ratio | 1 | 1 | 12.89904 | 21.3686 | <.0001* |  |
| Power | 1 | 1 | 14.21682 | 23.5516 | <.0001* |  |
| Time | 1 | 1 | 140.99002 | 233.5643 | <.0001* |  |
| Ratio*Ratio | 1 | 1 | 3.98476 | 6.6012 | 0.0146* |  |
| Ratio*Power | 1 | 1 | 0.00334 | 0.0055 | 0.9411 |  |
| Power*Power | 1 | 1 | 0.02698 | 0.0447 | 0.8338 |  |
| Ratio*Time | 1 | 1 | 7.90271 | 13.0916 | 0.0009* |  |
| Power*Time | 1 | 1 | 11.35044 | 18.8032 | 0.0001* |  |
| Time*Time | 1 | 1 | 0.17196 | 0.2849 | 0.5969 |  |

**Residual by Predicted Plot**

**Ratio**

**Leverage Plot**

**Power**

**Leverage Plot**

**Time**

**Leverage Plot**

**Ratio*Ratio**

**Leverage Plot**

**Ratio*Power**

**Leverage Plot**

**Power*Power**

**Leverage Plot**

**Ratio*Time**

**Leverage Plot**

**Power*Time**

**Leverage Plot**

**Time*Time**

**Leverage Plot**

Response Surface

| Coef | **Ratio** | **Power** | **Time** | **V 583** |
| --- | --- | --- | --- | --- |
| Ratio | -0.599779 | 0.0001043 | 0.2705056 | 0.7331167 |
| Power | . | -1.928e-6 | 0.0020262 | 0.0048103 |
| Time | . | . | 0.013844 | 0.8079181 |

Solution

| **Variable** | **Critical Value** |
| --- | --- |
| Ratio | -0.100573 |
| Power | 296.00202 |
| Time | 1.9764345 |

Solution is a

SaddlePoint

Critical values outside data range

Predicted Value at Solution

1.2659974

**Canonical Curvature**

Eigenvalues and Eigenvectors

|  |  |  |  |
| --- | --- | --- | --- |
| Eigenvalue | 0.0424 | -0.0000 | -0.6283 |
| Ratio | 0.20605 | -0.00512 | 0.97853 |
| Power | 0.02365 | 0.99972 | 0.00025 |
| Time | 0.97826 | -0.02309 | -0.20611 |
|  |  |  |  |

**Prediction Profiler**
